# Prognostic factor analysis of oral and maxillofacial Langerhans cell histiocytosis based on clinical findings and tumour microenvironment

**DOI:** 10.1186/s12903-025-07638-z

**Published:** 2026-01-10

**Authors:** Masaru Ogawa, Satoshi Yokoo, Emi Saitou, Mai Seki, Takahiro Yamaguchi, Keisuke Suzuki, Hideharu Nakamura, Takaya Makiguchi

**Affiliations:** 1 Department of Oral and Maxillofacial Surgery, and Plastic Surgery, Gunma University Graduate School of Medicine, 3-39-22, Showa-Machi, Maebashi-City, Gunma, 370-8511 Japan; 2 Department of Diagnostic Pathology, Gunma University Graduate School of Medicine, 3-39-22, Showa-Machi, Maebashi-City, Gunma, 370-8511 Japan

**Keywords:** Langerhans cell histiocytosis, Oral and maxillofacial region, Prognostic factor, Regulatory T cell, M2 macrophage, Interleukin 10

## Abstract

**Background:**

This study aimed to investigate the clinical prognostic factors of oral and maxillofacial Langerhans cell histiocytosis (LCH-OMF) and the dynamics of regulatory T (Treg) cells and M2 macrophages.

**Methods:**

We retrospectively analysed nine patients who were definitively diagnosed with LCH-OMF and examined clinical factors including age, sex, disease type, lesion site, clinical findings, the presence or absence of central nervous system (CNS) risk lesions, other organ lesions, treatment methods and prognosis. Immunohistochemical and fluorescent immunohistochemical analyses were performed to investigate prognostic factors from a cell biological perspective regarding the mechanism of onset in these patients.

**Results:**

None of the nine patients followed the previously reported clinical prognostic patterns, and no patient with lesions in cranio-maxillofacial bones within the CNS risk region developed CNS-related disease. One patient had multi-system LCH with risk organ involvement (MS [RO +]) and the poorest prognosis; in this case, an increase in Tregs in the LCH lesion may have caused tumour immunosuppression, suggesting an association with disease severity. Findings from this patient suggested that interleukin (IL)-10 secretion by M2 macrophages may be an initiating factor in the mechanisms that regulate tumour growth; however, this interpretation is hypothesis-generating and based on a small number of cases.

**Conclusion:**

Assessing the prognosis of LCH-OMF requires a comprehensive consideration of the disease type, age, CNS-risk regions, risk organs, acute systemic inflammatory response, and skin involvement. A better understanding of IL-10 derived from Tregs and M2 macrophages in LCH-OMF and in LCH overall may enhance our comprehension of inflammatory dysregulation and Langerhans cell progression in LCH and could help to identify potential treatment strategies.

## Background

Langerhans cell histiocytosis (LCH) is a disease in which Langerhans cells with immature dendritic cell characteristics from the bone marrow proliferate in various organs, causing tissue damage [1]. Whether LCH is an inflammatory disease or a tumour has been debated for several years. Findings such as cytokine storms in LCH lesions, evidence of viral involvement, and spontaneous regression in some cases support an inflammatory disease. In 2010, BRAFV600E, an oncogenic mutation, was identified in the LCH cells of approximately half of the patients. In addition, hyperphosphorylation of the extracellular signal-regulated kinase protein has been reported in almost all patients with LCH, even in those without a BRAF mutation. These results indicated that BRAF mutations are driver mutations in LCH. Mutations arising at the dendritic progenitor cell stage are associated with high-risk LCH, whereas mutations at the differentiated dendritic cell stage are associated with low-risk LCH. In other words, oncogenic genetic mutations occur in bone marrow progenitor cells. Additionally, viral infections and genetic polymorphisms contribute to these phenomena, leading to the abnormal proliferation of LCH cells. This results in the excessive secretion of cytokines/chemokines (cytokine storm) and the activation of osteoclasts, which are believed to contribute to tissue destruction. These observations led to the concept of LCH as an inflammatory myeloid neoplasm [2–7].

The main effector cells in the tumour microenvironment (TME) are the cytotoxic T (Tc) cells (CD8 + T cells) (cancer-specific CD8 + T cells). Additionally, other cells present include natural killer (NK) cells, γδT cells, and NK T cells, which are expressed at different times and localise in various areas. Activated Tc cells and NK cells express the chemokine receptor CXCR3, which recognises the chemokines CXCL9, CXCL10 and CXCL11 secreted by cancer cells and stromal immune cells such as M2 macrophages and vascular endothelial cells. As a result, activated Tc cells and NK cells accumulate in the TME and destroy cancer cells. Regulatory T (Treg) cells, myeloid-derived suppressor cells, M2 macrophages and regulatory B cells are immunosuppressive cell populations that negatively regulate cancer immunity.

In addition, many checkpoint molecules and metabolic substances induce T-cell immune tolerance to cancer antigens. These suppressor cells are induced in the TME by the cytokines and chemokines produced by cancer cells. Several reports exist on LCH cases in the oral and maxillofacial regions (LCH-OMF) [8–13]; however, few studies have comprehensively analysed multiple cases [14–16]. Only Zhang et al. [16] examined the pathology of LCH in the maxillofacial region from the perspective of TME.

This study aimed to investigate the clinical prognostic factors of LCH-OMF and the dynamics of Treg cells and M2 macrophages, which are immunosuppressive in the TME. Investigating the proliferation of LCH-OMF from the perspective of the TME is meaningful because it may help clarify the pathology of clinical progression and predict and understand the disease condition based on the biopsy specimen.

## Patients and methods

### Patients

This study conformed to the Declaration of Helsinki and was approved by the Institutional Research Committee of Gunma University (IRB number: HS2024-260). Written informed consent was obtained from all the enrolled patients and patients' parents using the opt-out method.

We included nine patients definitively diagnosed with LCH-OMF at the Department of Oral and Maxillofacial Surgery, Gunma University Hospital, between January 2010 and December 2022. In these nine patients with LCH-OMF, we clinically examined factors such as age, sex, disease type, lesion site, clinical findings, the presence or absence of central nervous system (CNS) risk lesions [17], other organ lesions, treatment methods, and clinical outcome. Given the small sample size and the heterogeneity of clinical courses, no formal survival analysis was performed; instead, clinical outcomes were described.

### Immunohistochemical and fluorescent immunohistochemical analysis

**Fig. 1:**
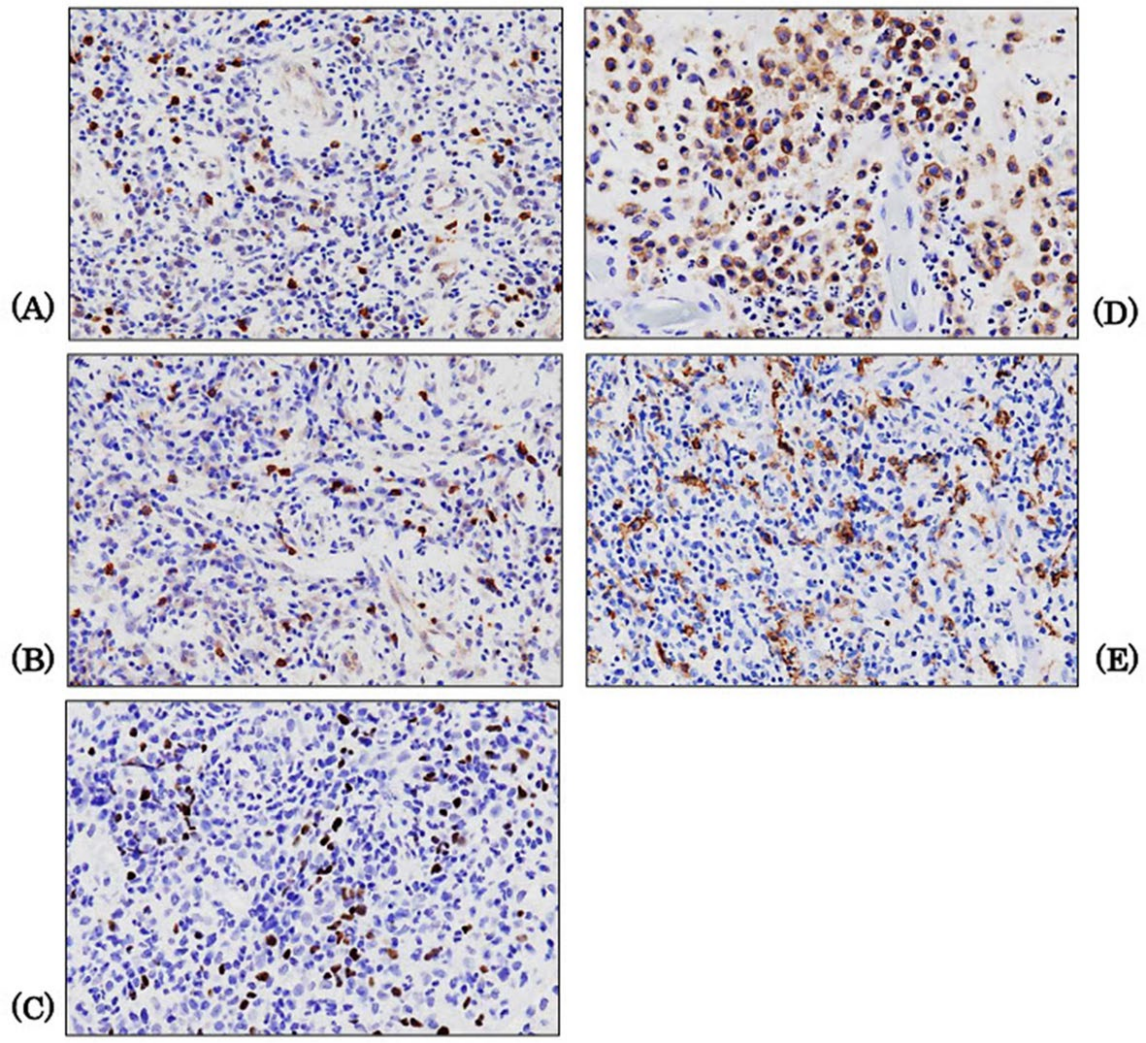
Immunohistochemical analysis. Immunohistochemical analysis is performed using the biopsy tissues to confirm the diagnosis of LCH in nine patients: The antibodies use included those targeting CD8(+) T cell (**A**), CD4(+) T cell (**B**), Foxp3(+) T cell (**C**), CD1a(+) Langerhans cell (**D**), and CD163(+) M2 macrophage (**E**). (400 × magnification). This figure shows the findings for Patient 9. LCH: Langerhans cell histiocytosis

An immunohistochemical analysis was performed on the biopsy tissue that was used to make a definitive diagnosis of LCH in nine patients. The primary antibodies and dilutions used in this study are listed in Table 1. FOXP3 was manually reacted overnight, and other antibodies (CD4, CD8, CD1a, CD163) were incubated with Leica BOND MAX (Leica Biosystems, Melbourne, Australia) at a 1:100 dilution for 15 min at room temperature. Visualisation was performed using 3,3'-diaminobenzidine tetrahydrochloride (DAB) to produce a brown colour, followed by counterstaining with haematoxylin. First, images of positive hotspots were captured using a BX-3 microscope and a DP-22 camera (Olympus, Tokyo, Japan) at 400 × magnification across the three fields of view. Subsequently, these images were analysed using WinROOF version 7.2 (Mitani-shoji LTD, Fukui, Japan). The number of positive cells was measured manually, and their mean values were calculated. FOXP3(+) cells were identified based on the number of DAB(+) nuclei. Cells with the DAB(+) cytoplasm were counted for other antibodies, and the apparent endothelial cells were excluded. For CD1a and CD163 staining, nuclei were counted as positive cells when the cytoplasm was dendritic or spindle-shaped and cell boundaries were unclear (Fig. 1).

**Table 1:** List of primary antibodies

**Antigen**	**Clone (Lot. No.)**	**Dilution**	**Source**
CD4	4B12 (NCL-L-CD4-368)	100	Leica
CD8	4B11 (NCL-L-CD8-4B11)	100	Leica
CD1a	MTB1 (NCL-L-CD1a-235)	100	Leica
CD163	10D6 (NCL-L-L-CD163)	100	Leica
		1000 (double staining)	
FOXP3	236A/E7 (#14–4777)	100	eBioscience
IL17A	Poly (GTX133781)	500	GeneTex
IL10	Poly (GTX130513)	500	GeneTex
STAT3	Poly (10253-2AP)	200	Proteintech
p19^INK4d^	Poly (10272-2AP)	200	Proteintech
Ki-67	Poly (NCL-Ki67p)	2000	Leica

**Fig. 2:**
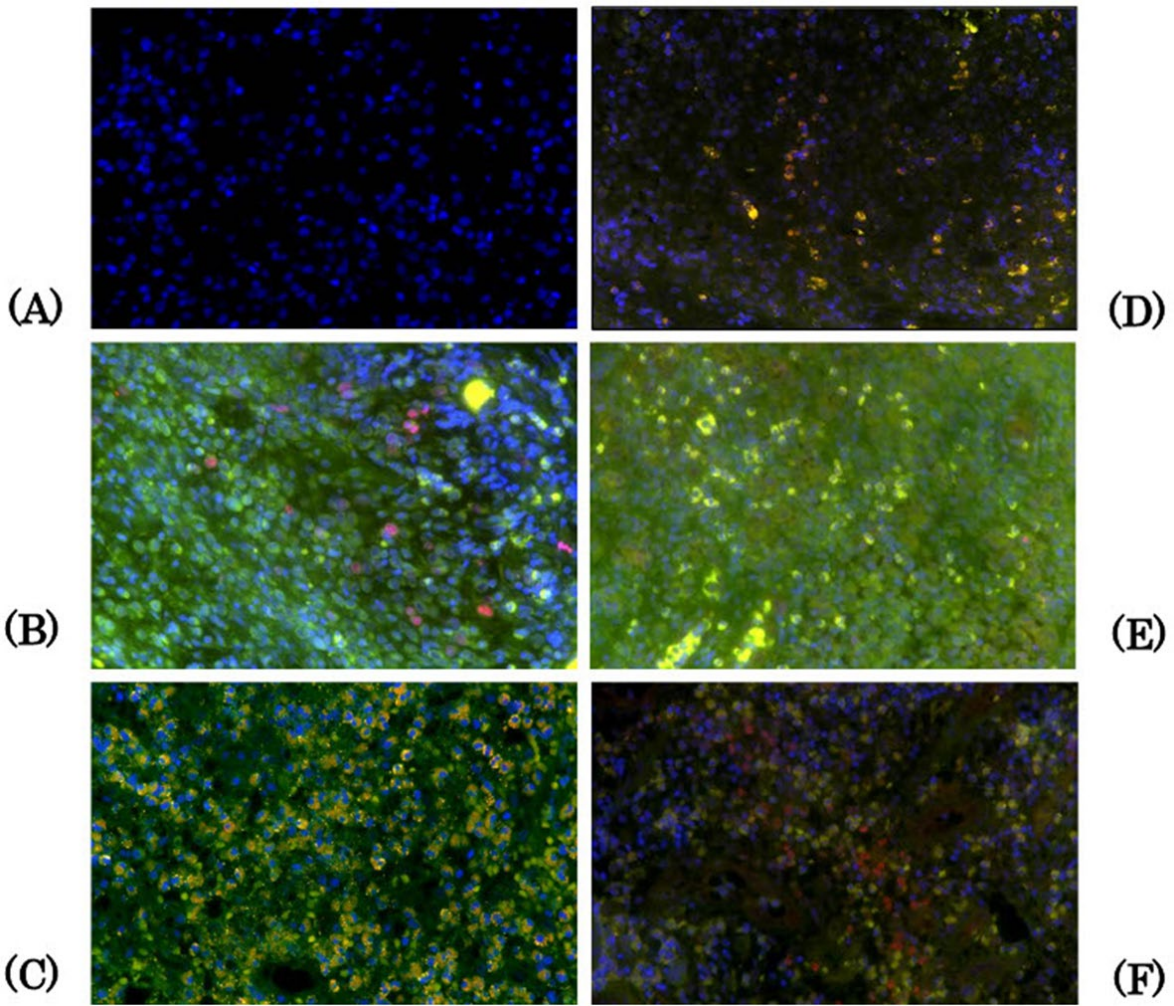
Double fluorescent immunohistochemical analysis. Nuclei are stained blue by reacting with DAPI for contrast staining (**A**). In the double staining of CD1a-Ki67 (**B**), the nuclei are red or a mixture of red and blue of DAPI (peach to reddish-purple). Cells showing green or yellow-green staining around the nucleus are considered co-positive cells. In other double immunostaining for CD4-IL-17A (**C**), CD163-IL10 (**D**), CD1a-STAT3 (**E**), and CD1a-p19INK4D (**F**), cells exhibiting a combination of green and red in the cytoplasm, or mixed colours from yellow-green to yellow and orange, are defined as co-positive. The number of nuclei is counted as the number of cells if the boundaries of the stained cells are unclear. Clear endothelial cells are excluded from the measurements. (400 × magnification). This figure shows the findings for Patient 9. DAPI:4', 6-diamidino-2'-phenylinole, dihydrochloride, IL: interleukin

IL-10 signaling is known to activate STAT3 and that STAT3 in turn induces p19INK4D as a cyclin-dependent kinase inhibitor in histiocytic cells, as reported previously [18, 19]. Therefore, this study conducted double immunostaining for CD4-IL17A, CD1a-Ki-67, CD163-interleukin (IL)−10, CD1a-STAT3, and CD1a-p19INK4D. The primary antibodies and dilutions used in the single immunohistochemical analyses were the same. The secondary antibodies for CD1a, CD4, and CD163 were fluorescently labelled in green using Alexa Fluor 488 chicken anti-rabbit IgG (A21200, Invitrogen, Carlsbad, CA, USA), and Ki-67, IL-10, IL-17A, STAT, and p19INK4D were labelled in red using Alexa Fluor 594 chicken anti-rabbit IgG (A21442, Invitrogen). Ki-67 was expressed in the nucleus, whereas the other proteins were expressed in the cytoplasm. All secondary antibodies were diluted to 1:500 and incubated at room temperature for 60 min. For contrast staining, the nuclei were stained blue by reacting with 4',6-diamidino-2'-phenylinole, dihydrochloride (DAPI) (VE063, Dojindo, Mashiki, Japan) for 10 min at room temperature. In the first stage of the measurement procedure, images of double-positive hotspots were taken with a fluorescence microscope (BZ-X810 KEYENCE, Osaka, Japan) at 400 × magnification in three fields of view, and merged images were created. In the next stage, these merged images were examined using WinROOF version 7.2 (Mitani-shoji LTD); double-positive cells were manually measured, and their mean values were calculated. Single-channel images were used if the merged images were unclear. The criteria for judgment were as follows: in the double staining of CD1a-Ki67 (A), the nuclei were red or a mixture of red and blue of DAPI (peach to reddish-purple), and cells that showed green or yellow-green staining around the nucleus were defined as co-positive cells. In the other double immunostaining of CD4-IL17A (B), CD163-IL-10 (C), CD1a-STAT3 (D), and CD1a-p19INK4D (E), cells exhibiting a combination of green and red in the cytoplasm, or mixed colours from yellow-green to yellow and orange, were defined as co-positive. The number of nuclei was counted as the number of cells if the boundaries of the stained cells were unclear. Clear endothelial cells were excluded from analysis. Two oral and maxillofacial surgeons and one pathologist performed these analyses (Fig. 2).

### Expression of FOXP3(+) cells and CD163(+) cells in each case of LCH-OMF

To investigate the relationship between tumour immune activity by CD8(+) T cells and the immune tolerance by Tregs, Foxp3(+) cell/CD4(+) T cells, Foxp3(+) cell/CD8(+) cells, and FOXP3(+) cell/CD4(-) IL17A(+) cell ratios were calculated for each patient.

### Influence of M2 macrophages on TME in LCH-OMF lesions

First, to clarify the relationship between M2 macrophage-derived IL-10 and LC proliferation, the correlation between IL-10-positive CD163 cells and Ki-67(+) CD1a cells was assessed in nine cases. Next, the effect of M2 macrophage-derived IL-10 on STAT3 expression in Langerhans cells (the ratio of STAT3(+) CD1a cells to IL-10(+) CD1a cells) was analysed. Third, the effect of STAT3 on p19^INK4D^ expression on Langerhans cells (the ratio of STAT3(+)CD1a cells to p19^INK4D^(+)CD1a cells) was investigated. Finally, the relationship between p19^INK4D^ expression and LCH proliferative activity (the ratio of p19^INK4D^(+)CD1a cells to Ki-67(+) CD1a cells) was examined. Linear regression analysis was performed for each independent and dependent variable in nine cases, and the regression coefficient (r) and its significance level (p-value) were calculated. Correlations were defined as strong when *r* ≥ 0.7, moderate when 0.4 ≤ *r* < 0.7, and weak when *r* < 0.4. Statistical analyses were performed using SPSS for Windows, version 25 (IBM Corp., Armonk, NY, USA). *P*-values < 0.05 were considered statistically significant.

## Results

### Analysis of patients’ clinical data

The clinical data of the nine patients are summarised by disease type in Table 2. Five of the nine patients were children aged 2–10 years and four were adults aged 25–65 years. The man-to-woman ratio was 4:5. Of the nine patients, three had single-system single-site (SS-s) LCH, two had single-system multiple-site (SS-m) LCH, and four had multi-system (MS) LCH. In one case of MS-LCH (Patient 9), a lung lesion, one of the at-risk organs, was observed (RO +). Two of the nine patients (Patients 6 and 8) had lesions in the maxillary bone, which were CNS-risk lesions. In Patient 6, two CNS-risk lesions were found in the maxillary and temporal bones. However, CNS lesions did not develop in either patient. In contrast, Patient 9 had developed central diabetes insipidus, although the affected craniofacial bones did not have CNS-risk lesions. Mandibular osseous lesions were observed in seven patients (Patients 1–5, 7, and 9). All patients except Patient 4, primarily complained of oral swelling and pain, which resulted from maxillary or mandibular osseous lesions. Only Patient 1 experienced a fever exceeding 38 °C, whereas four patients (Patients 2, 5, 8, and 9) reported fevers close to 38 °C. Five patients (Patients 1, 6, 7, 8, and 9) had an increased white blood cell count, and five patients (Patients 1, 2, 5, 8, and 9) had increased C-reactive protein (CRP) levels. Two of the four MS-LCH patients (Patients 6 and 8) had skin lesions. Only the patient with MS-LCH (RO +) died, owing to intestinal necrosis caused by aortitis syndrome.

**Table 2:** Patient characteristics

Patient	Age	Sex	Affected craanial and facial bone	Symptoms		CNS risk (onset)	Other-organ lesions	Treatment	Follow-up and prognosis
				Oral	Acute systemic inflammatory response				
SS-s LCH									
1	3Y	W	Mandible	Swelling Pain	Increased body temperature (38.0°C) Increased WBC count Increased CRP level	-	-	Observation after biopsy	5Y NED
2	65Y	W	Mandible	Swelling Pain	Increased body temperature (37.6°C) Increased CRP level	-	-	1st-Surgery 2nd-Surgery and RT (recurrence after 9M)	10Y NED
3	25Y	M	Mandible	Swelling Pain	None	-	-	Surgery	5Y NED
SS-m LCH									
4	56Y	W	Mandible	None	None	-	Bony pelvis	Chemotheraphy	12Y NED
5	10Y	M	Frontale Parietale Mandible	Swelling Pain	Increased body temperature (37.8°C) Increased CRP level	◯ -	-	Chemotheraphy	1Y5M NED
MS-LCH (RO-)									
6	10M	W	Maxilla Temporalis Occipitale	Swelling	Increased WBC count	◯ -	Skin (body, lower limb)	Chemotherapy	5Y NED
7	2Y	W	Mandible	Swelling Pain	Increased WBC count Increased lymphocyte count	-	Cervical lymph node	Surgery and chemotherapy	8Y NED
8	1Y	M	Maxilla	Swelling	Increased body temperature (37.5°C) Increased WBC count Increased CRP level Increased Monocyte count Increased lymphocyte count IL-2R up	◯ -	Skin (body lower limb) Rib Anus	Chemotheraphy	1Y8M NED
MS-LCH (RO+)									
9	57Y	M	Mandible	Swelling Pain	Increased body temperature (37.6°C) Increased WBC count Increased CRP level	- ◯ (CDI)	Pituitary gland lung Hypophysis	1st-Surgery 2nd-Surgery (recurrence after 3M) 3nd-RT (recurrence after 6M secondary surgery)	4Y10M DOD Intestinal necrosis (aortitis syndrome)

*CNS* Central nervous system, *WBC *White blood cell, *CRP* C-reactive protien, *RT *Radiation theraphy, *SS-s *Single-system single-site, *SS-m *Single-system multiple-site, *MS *Multi-system, *RO* Risk organ

### Expression of FOXp3(+) cells and CD163(+) cells on each case of LCH-OMF

**Fig. 3:**
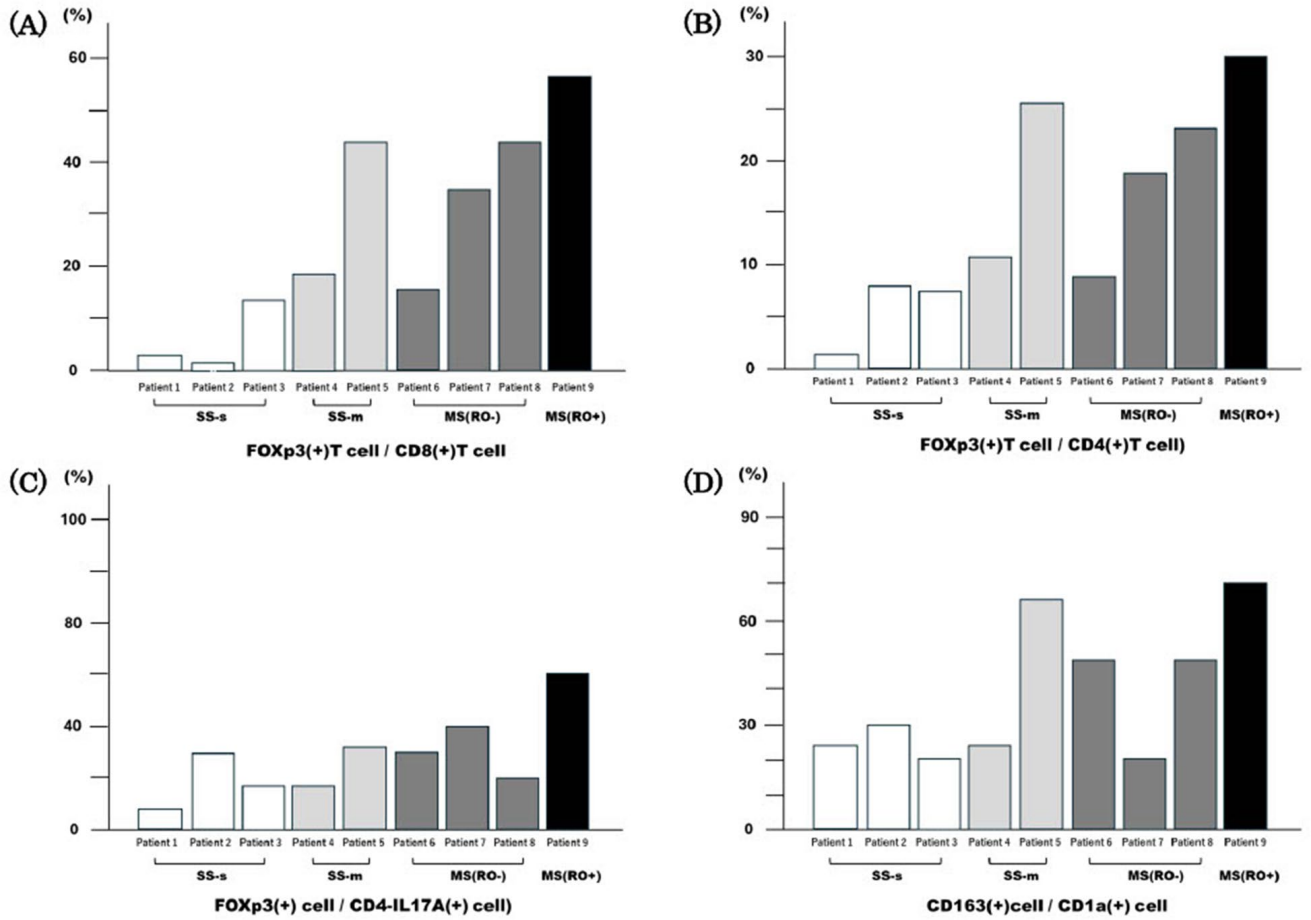
Expression of FOXP3(+) cells and CD163(+) cells on each case of LCH-OMF. In Patient 9 with MS (RO +), the expression ratio of Foxp3(+) cells to CD8(+) cells (**A**), Tregs to CD4(+) T cells (**B**), the expression ratio of Th17 (CD4-IL-17A) cells (**C**), and the density of M2 macrophages to LC cells, i.e. the CD163(+) cell/CD1a(+) cell ratio, is the highest (**D**). SS-s: single-system single site, SS-m: single-system multi-site, MS: multi-system, RO: risk organ, Treg: regulatory T cell, Th: helper T cell

In Patient 9, MS (RO +), the expression ratio of Foxp3(+) cells to CD8(+) cells was the highest (Fig. 3A). The expression ratio of Tregs to CD4(+) T cells was also the highest in Patient 9 (Fig. 3B), and the expression ratio of Th17 cells was the highest (Fig. 3C). The density of M2 macrophages to Langerhans cells in each patient and the CD163(+) cell/CD1a(+) cell ratio were calculated. Patient 9 had the highest expression ratio (Fig. 3D).

### Influence of M2 macrophages on TME in the cases of LCH-OMF lesions

**Fig. 4:**
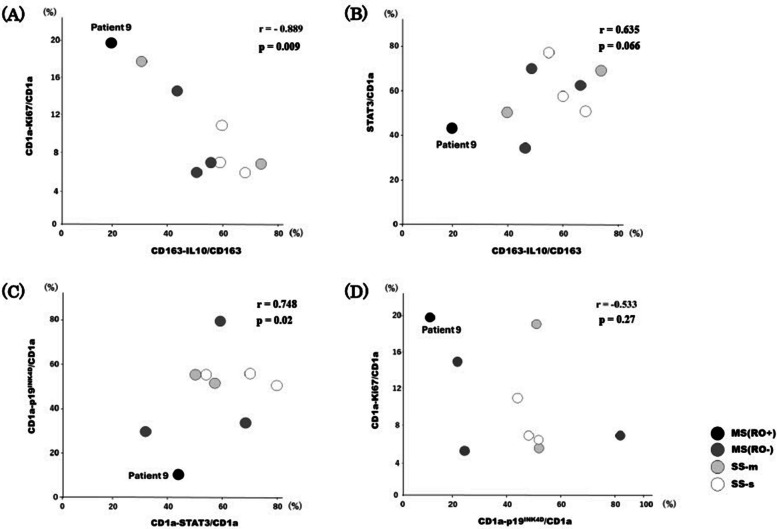
Influence of M2 macrophages on the TME in the cases of LCH-OMF lesions. LCH cells appear to have a high proliferative potential in patients with low IL-10 expression rates in M2 macrophages (*r* =—0.889, *p* = 0.089). Patient 9 with MS (RO +), characterised as the most severe disease type, exhibits the lowest IL-10 expression and highest proliferative potential (**A**). Additionally, the lowest IL-10 and STAT3 expression levels are also the lowest (**B**). When STAT3 expression is low, p19^INK4D^ expression in Patient 9 is reduced compared with that in the other cases (**C**), and Patient 9 displayed decreased p19^INK4D^ expression despite high LCH proliferative activity (**D**). LCH-OMF: oral and maxillofacial Langerhans cell histiocytosis, L: interleukin, SS-s: single-system single-site, SS-m: single-system multi-site, MS: multi-system, RO: risk organ

In the analysis of nine cases, a lower IL-10 expression rate in M2 macrophages was significantly associated with higher proliferative activity of Langerhans cells (*r* =—0.889, *p* = 0.0089). Among them, Patient 9, diagnosed with MS (RO +) and exhibiting the most severe disease phenotype, demonstrated the lowest IL-10 expression and the highest proliferative potential (Fig. 4A). Patient 9 also had the lowest IL-10 expression, which was accompanied by the lowest STAT3 expression. A tendency toward correlation was observed between IL-10 expression levels in M2 macrophages and STAT3 expression in Langerhans cells, although it did not achieve statistical significance (*r* = 0.635, *p* = 0.066) (Fig. 4B). In Langerhans cells (CD1a (+) cells), the expression levels of STAT3 and p19^INK4D^ showed a significant strong correlation (*r* = 0.748, *p* = 0.02). When STAT3 expression was low, p19^INK4D^ expression was also low (Fig. 4C). Patient 9 showed reduced p19^INK4D^ expression despite high LCH proliferative activity (CD1a-Ki67(+)/CD1a) (Fig. 4D). In the nine cases examined, a moderate correlation was observed, but it did not achieve statistical significance (*r* = −0.533, *p* = 0.27).

### Case presentation of MS(RO +) (Patient 9)

**Fig. 5:**
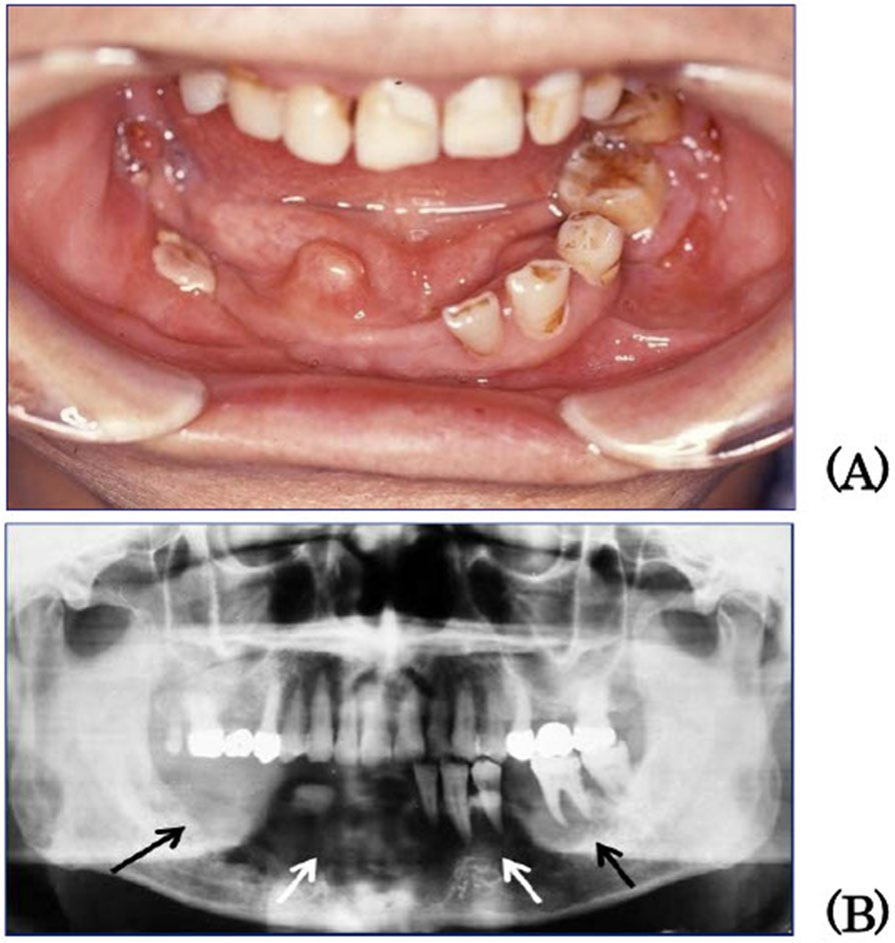
Patient 9. A 57-year-old man experienced respiratory impairment caused by pulmonary LCH, as well as renal dysfunction resulting from posterior pituitary LCH and aortitis syndrome. Swelling and tenderness of the right mandible and gingival swelling from the right second molar to the left second molar were noted, and the remaining teeth were unstable (**A**). The panoramic radiograph revealed bone absorption in the mandibular body, extending from the right second molar to the left second molar (**B**). This patient had MS(RO +) of LCH, a progressive disease. LCH: Langerhans cell histiocytosis, MS: multi-system, RO: risk organ

A 57-year-old man was admitted to the Department of Internal Medicine, Gunma University Hospital in January 2010 for respiratory impairment due to pulmonary LCH and renal dysfunction owing to posterior pituitary LCH and aortitis syndrome. In June 2010, the patient developed a swelling of the right mandible and was referred to the Department of Oral and Maxillofacial Surgery. Swelling and tenderness of the right mandible and gingival swelling from the right second molar to the left second molar were noted, and the remaining teeth were unstable (Fig. 5A). No trismus or sensory abnormalities were recognised in the area innervated by the inferior alveolar nerve. A panoramic radiograph showed bone absorption in the mandibular body from the right second molar to the left second molar (Fig. 5B). The biopsy result indicated a pathological diagnosis of mandibular LCH. In September 2010, extirpation and curettage were performed under general anaesthesia. The lesion recurred in September 2011, and extirpation and curettage were performed again; however, recurrence was observed again in July 2012. As respiratory function was considered compromised due to progressive pulmonary LCH, 20 Gy external beam radiotherapy was administered instead of surgery. Subsequently, the mandibular LCH did not recur, and pulmonary LCH was treated with steroids and smoking cessation. He died in May 2015 owing to intestinal necrosis caused by aortitis syndrome. Aortitis syndrome is thought to involve immune abnormalities, as many immune cells, such as macrophages and T cells, are found in vasculitis tissues, and immunosuppressive therapy often shows positive results. The relationship between LCH and aortitis syndrome in this patient remains unclear. As this patient had MS(RO +) of LCH, a progressive disease, it is thought that the activity of effector cells in the TME increased, resulting in a more vigorous immune response. The combination of pulmonary lesion associated with aortitis syndrome and central diabetes insipidus caused by posterior pituitary LCH was assumed to lead to deterioration of the patient's general condition. Although the affected craniofacial bones were not CNS-risk lesions, this patient had developed central diabetes insipidus.

## Discussion

In this study, we made two important clinical observations. First, the nine patients presented did not clearly follow previously reported clinical prognostic pattern. Second, none of the patients with lesions in craniomaxillofacial bones, which are considered CNS risk regions, developed CNS-related disease.

Although predicting the prognosis of LCH is challenging, the following predictive factors have been reported: (1) disease type, that is, the site of the lesion, (2) age < 2 years and (3) severity of functional impairment at the lesion site [18, 19]. Kobayashi et al. [5] found that the presence of initial fever and skin involvement is associated with a refractory course of LCH under current treatment strategies. In this study, as demonstrated by Kobayashi et al. [5], long-term survival of over 5 years was observed in patients with LCH-OMF who did not have an acute systemic inflammatory response. On the other hand, patients with SS-s survived for over 5 years, even in the presence of an acute systemic inflammatory response. Patients with MS (RO-) survived for over 5 years, even though primary skin lesions suggested as refractory factors were observed. Only patients with MS (RO +) who exhibited an acute systemic inflammatory response had a poor prognosis within 5 years. Kobayashi et al. [5] pointed out that the magnitude of the association between LCH prognosis and primary skin lesions was not substantial when considering a wide confidence interval. Therefore, it is important to consider these three established clinical factors together when predicting prognosis in LCH [20, 21].

The craniomaxillofacial bones are the most affected organs in LCH, and most parts of the bones are at risk of affecting the CNS [17]. The maxillary bone, zygomatic bone, and maxillary sinus require initial attention in the oral and maxillofacial regions. Three of the nine patients in this study had lesions in the maxillary bone, and one had lesions in both the maxillary and temporal bones. No CNS-related diseases occurred in any of the three patients. However, since oral and maxillofacial surgeons may initially examine LCH-OMF patients, it is essential to consider that craniomaxillofacial bone lesions are CNS-risk lesions.

We made two important experimental observations in terms of the TME in LCH-OMF. First, in Patient 9, who had the most advanced and poor prognosis of MS(RO +), the increase in Tregs in the LCH lesion may have caused tumour immunosuppression, suggesting an association with LCH severity. Second, based on a comprehensive assessment of nine cases, it is not inconceivable that IL-10 secretion by M2 macrophages constitutes the starting point of the tumour proliferation and inhibition mechanism. This characteristic was clearly observed in Patient 9 with MS (RO +).

Several studies have reported an increase in the number of FOXP3(+)Treg cells in LCH lesions [16, 22–27]. Zhang et al. [16] confirmed the expansion of the Treg population in response to the proliferation of LCH cells in LCH-OMS lesions. Tregs account for 5–10% of CD4(+) T cells in normal tissues [3, 22]. In Patient 9 with MS (RO +), Tregs represented over 30% and were extremely elevated compared to normal tissues. Considering the expression ratios of Tregs to CD8(+) T cells, CD4(+) T cells, and helper T (Th)17 cells in Patient 9, it is likely that the suppression of tumour immunity in the TME is related to the type and severity of LCH. Patient 5 had SS-m and given the low Treg/Th17 ratio and the dominance of Th17 cells at the time of treatment, this patient was also considered to have mild disease and a good prognosis. However, considering the three clinically affected cranial and facial bones, an acute systemic inflammatory response, high ratios of Tregs to CD8(+) and CD4(+) T cells and M2 macrophages to LC, and a short follow-up period of 1 year and 5 months, it was suggested that prognosis-affected lesions may occur in the future.

**Fig. 6:**
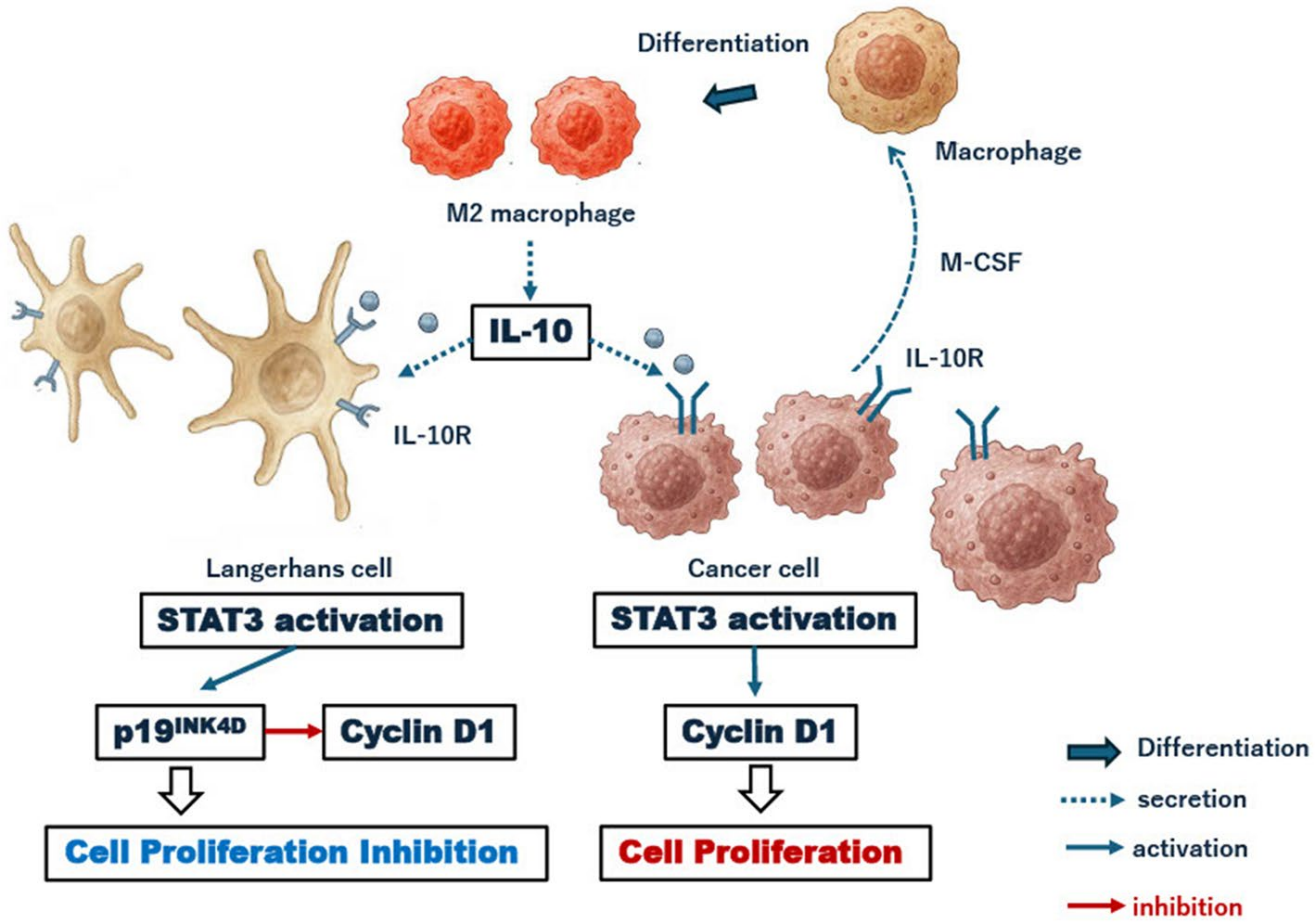
Mechanism of LCH cell suppression. Patient 9 exhibits the lowest IL-10 expression and the highest proliferation rate among all patients. The expression of STAT3, activated by IL-10, is low, and p19^INK4D^ expression, which is activated by STAT3, is the lowest. Decreased p19^INK4D^ expression leads to LC cell proliferation. STAT3, phosphorylated by IL-10, activates cell growth factors such as cyclin D1, thereby promoting cancer cell proliferation. However, in histiocytic cells such as Langerhans cells, phosphorylated STAT3 activates p19^INK4D^, a cyclin kinase inhibitor. This inhibits the activity of cell growth factors, and suppresses cell proliferation. LCH: Langerhans cell histiocytosis, IL: interleukin, IL-10R: interleukin 10 receptor, LC: Langerhans cell

Our data indicate that, in this small cohort of nine cases, IL-10 secretion by M2 macrophages may be the initial step in the processes that regulate tumour proliferation and inhibition, particularly in Patient 9 with MS (RO +). On this basis, we propose a hypothesis in which M2 macrophages are implicated in both tumour proliferation and inhibition. In various tumours, macrophages that infiltrate the tumour tissue differentiate into M2 macrophages, promoting tumour proliferation by suppressing tumour immunity and angiogenesis [28–30]. In gliomas, M2 macrophage density in the lesions correlates with tumour malignancy [28]. In breast and bladder cancers, IL-10 produced by M2 macrophages binds to IL-10 receptors on cancer cells, activating STAT3 and subsequently upregulating growth-related molecules, such as cyclin D1 by activating STAT3 [31–33]. This mechanism promotes cancer cell proliferation. Patient 9 exhibited the lowest IL-10 expression and the highest proliferation rate among all presented patients (Fig. 4A). In addition, the expression of STAT3, which is activated by IL-10, was low, and the expression of p19^INK4D^, activated by STAT3, was the lowest (Fig. 4B, C). Thus, a decrease in p19^INK4D^ expression may cause Langerhans cells proliferation (Fig. 4D). STAT3, phosphorylated by IL-10, activates cell growth factors such as cyclin D1, and causes cancer cell growth. In contrast, in histiocytic cells such as Langerhans cells, phosphorylated STAT3 activates p19^INK4D^, a cyclin kinase inhibitor. Consequently, the activity of cell growth factors is inhibited, suppressing cell proliferation [18, 19] (Fig. 6).

This overall pattern was observed across all nine cases and was most clearly illustrated in Patient 9, who had MS (RO +) with a poor prognosis. Analysis of all nine cases revealed a strong inverse correlation between IL-10 expression in M2 macrophages and LCH proliferative activity (r = − 0.889, p = 0.0089; Fig. 4A). Taken together, the findings on the influence of M2 macrophages on the TME in LCH-OMF lesions (Fig. 4B–D) suggest that IL-10 secreted by M2 macrophages may be involved in regulating LCH cell proliferation and growth inhibition. Furthermore, immunosuppression caused by the inactivation of effector T cells by IL-10 secreted from Tregs is well documented. This raises the possibility that IL-10 may behave similarly within the TME of LCH, influencing cell proliferation and inhibition.

This study has several limitations. First, it included only nine cases from a single centre, and therefore the findings should be regarded as exploratory and hypothesis-generating rather than definitive. Second, the cell biological examinations were limited to protein-level analyses using immunostaining; multi-centre collaborative research and additional molecular biological investigations will be acquired to validate and extend these observations. Third, throughout this study, we have mainly discussed the possibility that immune dysfunction may induce tumour proliferation, it is also plausible that tumour proliferation itself may induce or exacerbate immune dysfunction, and that further studies will be required to clarify this relationship.

In conclusion, assessing the prognosis of LCH-OMF requires a comprehensive consideration of the disease type, age, CNS-risk regions, risk organs, acute systemic inflammatory response, and skin involvement. A better understanding of IL-10 derived from Tregs and M2 macrophages in LCH-OMF and in LCH overall may enhance our comprehension of inflammatory dysregulation and Langerhans cell progression in LCH and could help to identify potential treatment strategies.

## Data Availability

The datasets generated during and/or analysed during the current study are available from the corresponding author on reasonable request.

## Abbreviations

LCH: Langerhans cell histiocytosis
TME: Tumour microenvironment
Tc: Cytotoxic T cell
Treg: Regulatory T cell
NK: Natural killer
OMF: Oral and maxillofacial
CNS: Central nervous system
DAB: 3,3'-Diaminobenzidine, tetra-hydrochloride
IL: Interleukin
DAPI: 4'-6-Diamidino-2'-phenylinole, dihydrochloride
SS-s: Single-system single-site
SS-m: Single-system multi-site
MS: Multi-system
RO: Risk organ
Th: Helper T cell
CRP: C-reactive protein
